# Impact of Intravenous Tranexamic Acid on Postoperative Complications in Gender-Affirming Mastectomy: A Focus on Drain Output and Duration

**DOI:** 10.1007/s00266-026-05797-0

**Published:** 2026-03-17

**Authors:** Lisa Radacher, Maximilian Zaussinger, Sandra Feldler, Bernhard Schwartz, Manfred Schmidt

**Affiliations:** 1 Medical Faculty, Johannes Kepler University Linz, Altenbergerstr. 69, 4040 Linz, Austria; 2 Department of Plastic, Aesthetic and Reconstructive Surgery, Kepler University Hospital, Krankenhausstr. 9, 4020 Linz, Austria; 3 Department of Research and Development, University of Applied Sciences for Health Professions Upper Austria, Semmelweisstr. 34/D3/2, 4020 Linz, Austria

**Keywords:** Tranexamic acid, Gender-affirming mastectomy, Drain output, Drain duration, Hematoma

## Abstract

**Background:**

Tranexamic acid (TXA) has been increasingly acknowledged as a beneficial pharmacological agent in plastic surgery. However, despite its proven efficacy and safety, there is limited research on its intravenous application in gender-affirming mastectomy, particularly regarding its effect on drain duration, drain output, and length of hospital stay.

**Methods:**

In this retrospective single-center study, patients who underwent double incision mastectomy with free nipple grafts were categorized into two cohorts: one group receiving TXA and a control group without TXA utilization (TXA vs no-TXA). Demographic characteristics, surgical data, and complication rates were analyzed and compared.

**Results:**

A total of 75 patients were included, with 34 procedures performed without the administration of TXA and 41 patients receiving TXA. The median age of the study population was 24 years (range 18–57). Postoperative bleeding requiring surgical revision was significantly less frequent in the TXA group (*p* = 0.038). Total drain output (*p* = 0.048), drain duration (*p* < 0.001) and length of hospital stay (*p* = 0.008) were significantly reduced in the TXA group. No thromboembolic events or seizures were observed.

**Conclusions:**

This study demonstrated that intravenously administered TXA significantly reduces the incidence of postoperative hematoma, length of hospital stay, drain duration, and output after gender-affirming mastectomy. Based on these findings, we would recommend the use of TXA in double incision mastectomy with free nipple grafts to minimize the occurrence of seroma formation and major bleeding-related complications.

**Level of Evidence IV:**

This journal requires that authors assign a level of evidence to each article. For a full description of these Evidence-Based Medicine ratings, please refer to the Table of Contents or the online Instructions to Authors  www.springer.com/00266.

## Introduction

Gender-affirming mastectomy, also known as top surgery or chest masculinization, is the most frequently performed surgical procedure in transgender males [1, 2]. It is often the primary or sole surgical intervention for individuals undergoing a medical transition process [3]. Top surgery plays an important role in addressing gender incongruence and has been shown to significantly improve quality of life [4, 5]. In contrast with female mastectomy procedures, the overall goal of top surgery is to mimic a more masculine chest phenotype, repositioning and resizing the nipple–areola complex (NAC) and sculpting the chest to achieve a more defined contour [6, 7]. To date, various algorithms exist to determine the most appropriate surgical technique based on factors such as preoperative breast size, severity of ptosis, and skin elasticity [6, 8, 9]. However, variations in the double incision mastectomy with free nipple grafts (DIFNG) remain the most applied approach [10].

Frequent complications reported in the literature after gender-affirming mastectomy include minor and major bleeding-related issues [11–13]. Interestingly, hematomas occur more frequent in gender-affirming mastectomy compared to other types of breast surgery, reporting incidences that reach up to 31.2% [12]. To counter these prevalent bleeding-related complications, TXA has become increasingly popular in the field of plastic surgery, as accumulating evidence highlights its effectiveness in different surgical procedures [14–19]. By competitively binding to lysine sites on plasminogen, TXA prevents its conversion to plasmin and thus inhibits fibrin degradation [20]. In addition, TXA reduces plasmin-mediated platelet activation, preserving platelet function and enhancing clot stability [21]. However, findings regarding the use of TXA in breast [15, 17, 18] and top surgery [22–24] remain inconsistent. The few existing studies vary in design and results [22–25], and topical applications in particular have not consistently demonstrated significant benefits in reducing bleeding or seroma related complications after gender-affirming mastectomy [22, 23, 25, 26]. Furthermore, drain duration and output following intravenous administration of TXA have not been well studied yet [26].

The aim of this study was to evaluate the safety profile of intraoperatively administered intravenous TXA and its potential effects on complication rates following top surgery, especially with respect to drain duration, total drain output, and length of hospital stay. To achieve this, the patient population was divided into two groups (TXA vs no-TXA). One group received intraoperative TXA and was compared to a control group without TXA administration. This study aims to provide a comprehensive understanding of whether the use of TXA influences recovery time and the risk of adverse events.

## Methods

This single-center retrospective cohort study was conducted in compliance with the local ethical standards and the Helsinki Declaration for the Ethical Treatment of Human Subjects. It was approved by the local review board (EK-No. 1124/2025). All patients provided written and verbal informed consent. The study included transmasculine patients aged above 18 years, who underwent DIFNG mastectomy between January 2018 and December 2024 and had a stable health condition. Patients who received intraoperative TXA formed the intervention group, and their outcomes were compared to a control group who underwent the same procedure during the same time period without TXA administration. Individuals undergoing gender-affirming mastectomy using alternative surgical techniques, such as periareolar incision, were excluded from our study. The administration of TXA was determined by the surgeon’s intraoperative assessment of an increased bleeding tendency, including diffuse intraoperative capillary oozing from the wound bed or persistent bleeding despite adequate hemostasis after tissue resection. TXA was administered intravenously using a bodyweight adapted dosage after tissue resection and before wound closure. In practice, administered doses ranged from approximately 1–2 g. TXA was administered as a single intraoperative intravenous dose only, with no postoperative continuation. No intraoperative redosing occurred. This single-center retrospective study was performed by four surgeons, all following the same standardized operative protocol and contributing to both groups (TXA and no-TXA).

Patients’ demographic characteristics, including age, sex, body mass index (BMI), smoking status, and comorbidities, were analyzed. Surgical data were collected, including the date of surgery, surgical technique, and resection weight. The main focus of the analysis was the observation of drain duration, drain output, and length of hospital stay. Drain output was recorded for the first three days and the total hospital stay. In addition, postoperative complications were recorded, with particular attention to hematoma requiring surgical revision and seroma formation. Seroma was defined as the development of clinical symptoms and ultrasound-detected fluid accumulation requiring percutaneous aspiration. Additionally, wound infection, wound dehiscence, and TXA-related adverse events were analyzed.

### Surgical Technique and Postoperative Care

**Fig. 1:**
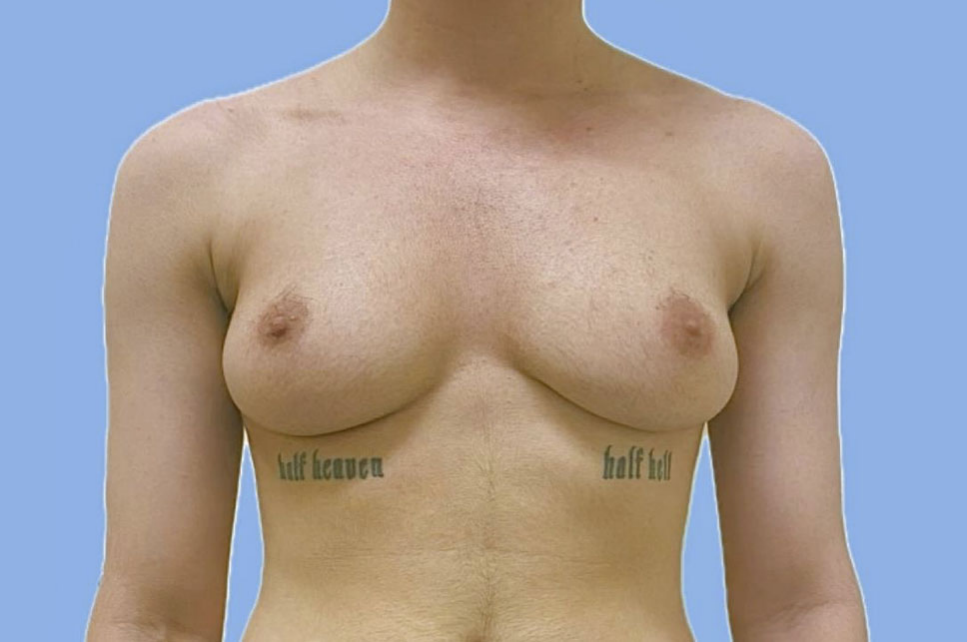
Preoperative frontal view of a 25-year-old transmasculine patient presenting with gender dysphoria and seeking chest masculinization. The patient was scheduled for double incision mastectomy with free nipple grafts (DIFNG)

**Fig. 2:**
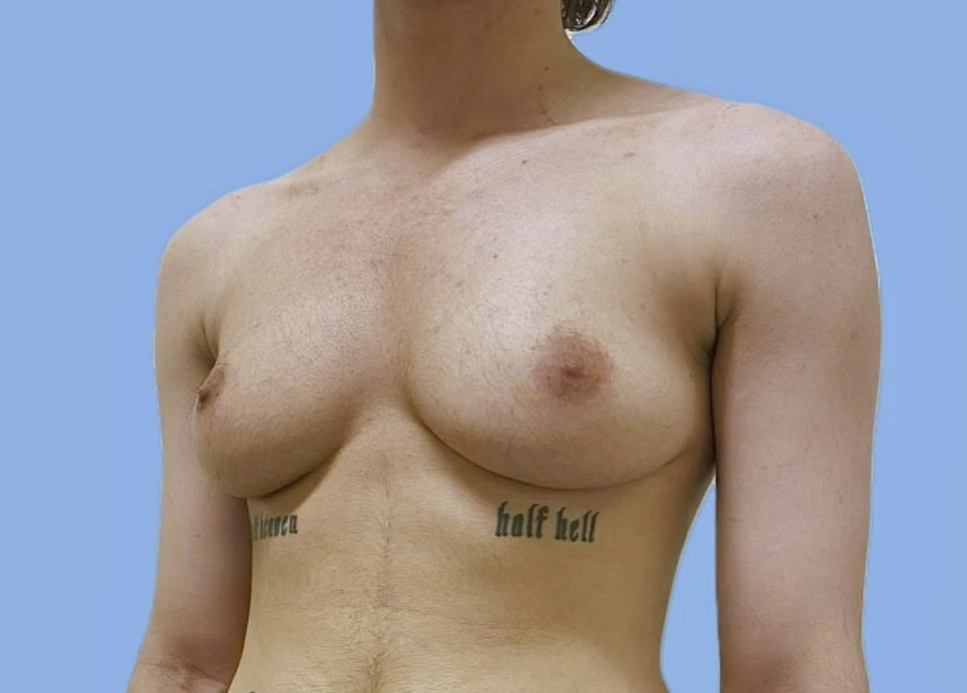
Preoperative 45-degree view of the 25-year-old transmasculine patient undergoing double incision mastectomy with free nipple grafts

**Fig. 3:**
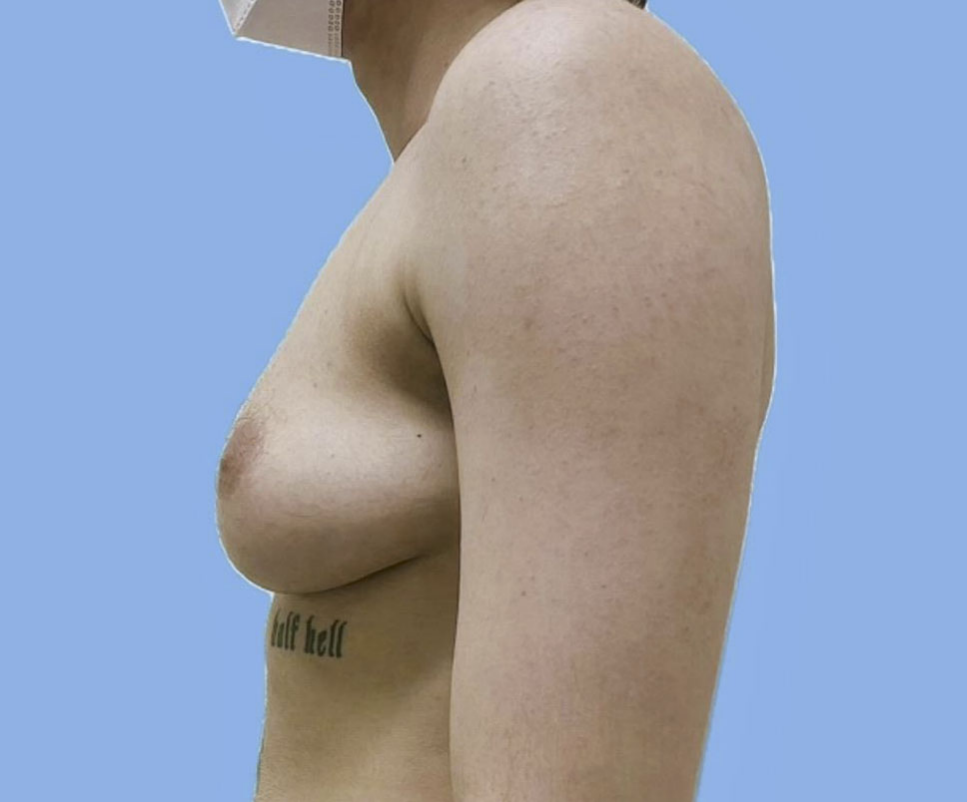
Preoperative lateral view of the same 25-year-old transmasculine patient prior to double incision mastectomy with free nipple grafts, demonstrating the native chest contour and breast projection

**Fig. 4:**
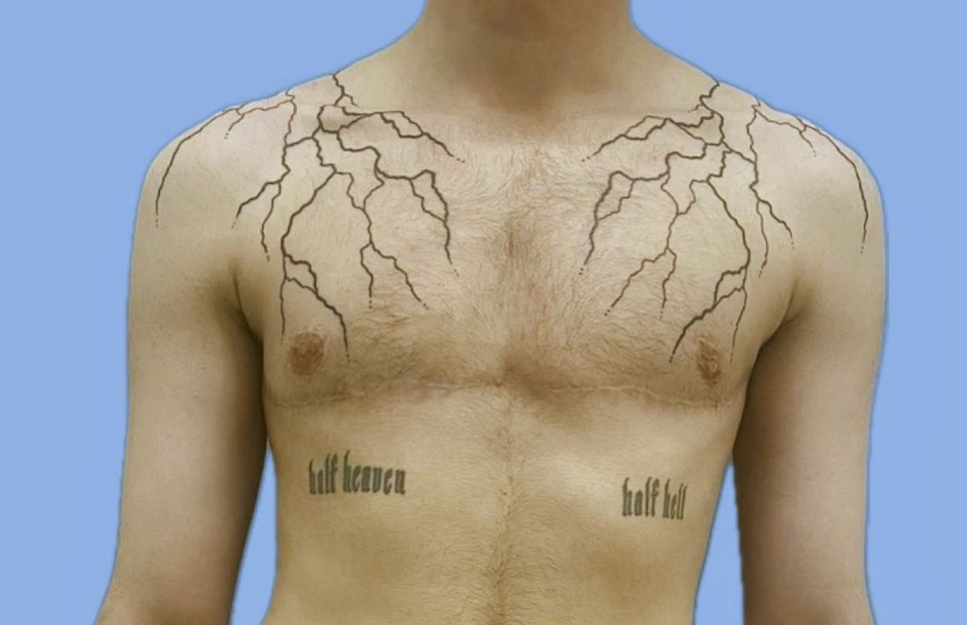
Postoperative frontal view of the same patient 12 months after a successfully performed double incision mastectomy with free nipple grafts, demonstrating a flat, masculine chest contour, and well-healed scars

**Fig. 5:**
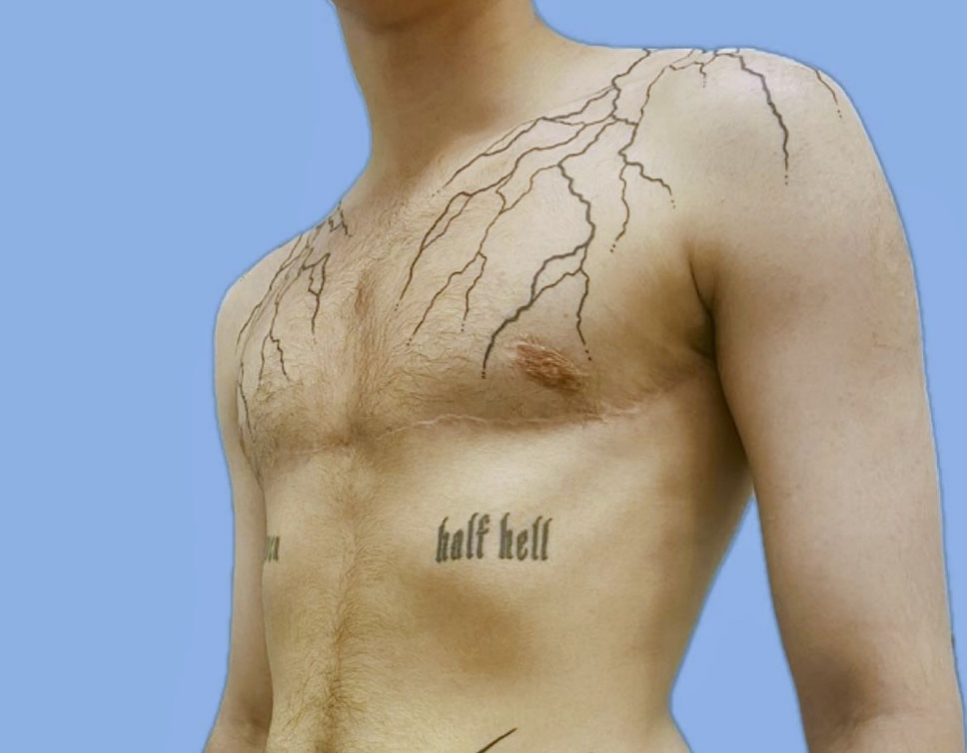
Postoperative 45-degree view 12 months after surgery, illustrating symmetric chest shape following double incision mastectomy with free nipple grafts

**Fig. 6:**
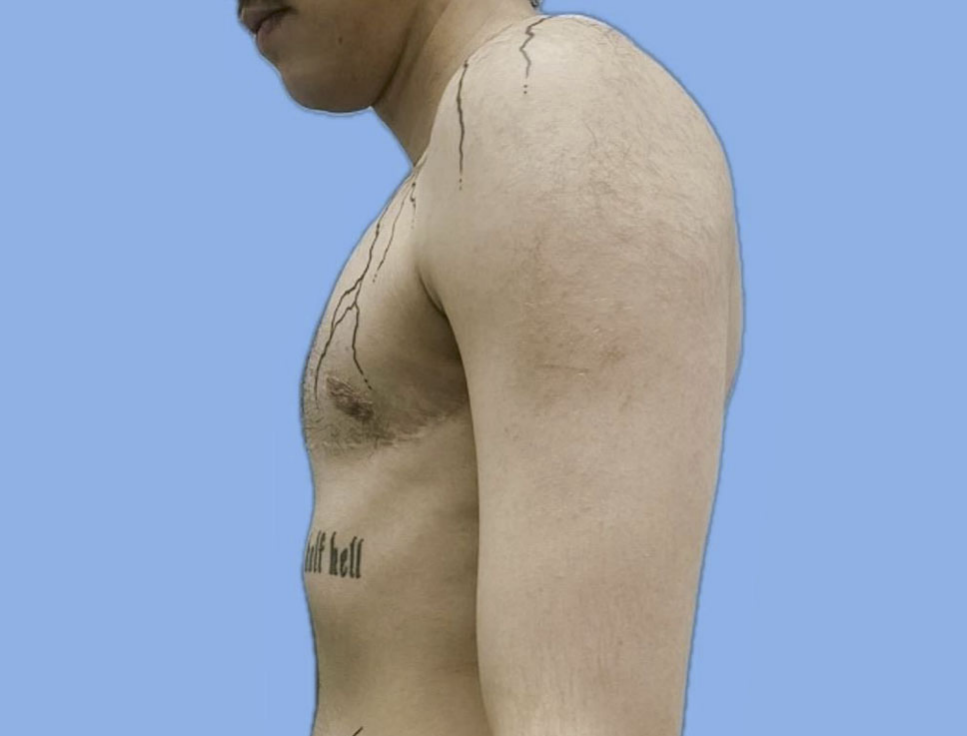
Postoperative lateral view of the 25-year-old transmasculine patient 12 months following double incision mastectomy with free nipple grafts, showing stable contour and good skin adaptation

The preoperative markings of the DIFNG mastectomy were performed with the patient in a standing position. The inferior margin of the planned incision was drawn in the original inframammary fold (IMF), while another line was marked above the NAC to define the superior margin, using a pinch test to ensure tension-free wound closure. Laterally, both incision lines were extended and connected toward the axilla, resulting in a future L-shaped scar. The procedure was carried out under general endotracheal anesthesia. The skin was incised with a scalpel, subsequent to the preparation with monopolar cautery (ValleyLab Force FX, Medtronic, Dublin Ireland) along the markings. The NAC was excised at full thickness, thinned, and prepared for free transplantation. Further preparation was performed using monopolar diathermy, dissecting down to the muscle fascia. The mastectomy flap was then dissected just below the Scarpa’s fascia toward the second rib. Laterally, beneath the border of the pectoralis major muscle, the dissection did not include the entire soft tissue down to the muscle fascia. The soft tissue, including the mammary gland and skin, was then dissected while preserving the pectoral muscle fascia. One 15-gauge suction drain (Blake drain 15 FR, Ethicon, Vienna, Austria) was placed on the lateral side of each breast. Quilting or progressive tension sutures were not utilized. The wound was closed in a 3-layered tension-free fashion, using an absorbable 3/0 braided suture for the deep dermis (Polysorb TM, Medtronic, Vienna, Austria), an absorbable 3/0 braided suture (Polysorb TM, Medtronic, Vienna, Austria) for the superficial dermis, and an absorbable 3/0 barbed intracuticular running suture (V-loc TM, Medtronic, Vienna, Austria). The neo-NAC position was placed at least 1.5 cm above the IMF scar and approximately 1–3 cm medial to the anterior axillary line. The neo-NAC position was then deepithelialized. The prepared NAC was inserted and secured circumareolarly using a single-layer wound closure with a non-absorbable 5/0 monofilament suture (Monosof TM, Medtronic, Vienna, Austria). Lomatuell wound dressing and antibiotic ointment were applied, followed by a bolster tie-over dressing above the NAC. Ultimately, a breast compression binder was applied. Intraoperatively applied compression binders were maintained until drain removal, followed by immediate use of a compression bra. Initial dressing changes were performed by the surgical team. Patients were instructed to continue compression therapy by wearing a compression bra for 6 weeks. The removed breast tissue was sent for histological examination. The drains were removed if the collected fluid was below 30 ml/24 h and earliest on the second postoperative day. The tie-over bolster dressing and the circumareolar sutures around the NAC were removed after 14 days. Strict avoidance of heavy lifting or exercising during the first 8 postoperative weeks was recommended. Preoperative photographs of a patient undergoing DIFNG mastectomy are shown in Figs. 1, 2 and 3. Figures 4, 5 and 6 illustrate the postoperative results following a successfully performed procedure.

### Statistical Analyses

All data were analyzed as available (i.e., no statistical imputation for missing data). Statistical analyses were performed using IBM SPSS Version 29.0 (SPSS Inc., Chicago, IL, USA). For analyzing between-group differences Mann–Whitney U-test as well as Fisher’s exact and Chi-squared tests were executed for metrical as well as nominal variables, respectively. For all statistical analyses, an alpha value of 0.05 was set to be acceptable.

## Results

### Demographic and Surgical Data

A total of 75 transmasculine patients fulfilled the above described inclusion criteria and were separated into two groups based on TXA utilization (TXA vs no-TXA). TXA was administered intravenously to 41 patients, while 34 patients did not receive TXA. Overall, the study cohort had a median age of 24 years (range 18–57 years), a median BMI of 24 kg/m^2^ (range 18–40 kg/m^2^), and a median follow-up period of 36 months (range 9–93 months). Comorbidities were present in 11 patients (14.7%), while 15 participants (20.0%) had a history of smoking. Demographic data did not differ significantly between groups, indicating a homogenous study population. Further details are demonstrated in Table 1. The median weight of resected tissue of both breasts combined was 980 grams (range 60–3320 g). Median postoperative drain output of the first three days was 140 ml (range 50–360 ml), and total median drain output was 150 ml (range 50–400 ml). Drains were removed after a median of 3 days (range 2–6 days), which aligned with a median hospital stay of 3 days (range 2–6 days). On average patients attended 3 follow-up appointments (range 1–5). Statistically significant group differences were observed regarding total drain output (*p* = 0.048), drain duration (*p* < 0.001), and length of hospital stay (*p* = 0.008). All surgical data are demonstrated in Table 2.

**Table 1:** Baseline demographic characteristics of patients undergoing double incision mastectomy with free nipple grafts (DIFNG), comparing those who received intraoperative tranexamic acid (TXA) with those who did not

	Total	TXA	No-TXA	*p* Value
No. of patients	75	41	34	–
Age at surgery, years	24.0 [18, 57]	24.0 [19, 42]	23.5 [18, 57]	0.987
BMI at surgery, kg/m^2^	24.0 [18, 40]	24.0 [18, 40]	24.0 [19, 39]	0.960
No. of comorbidities	14.7% (11)	14.6% (6)	14.7% (5)	0.622
No. of smoking history	20.0% (15)	17.1% (7)	23.5% (8)	0.341

Values are represented by median [min, max], percent (number)

Between-group differences were determined via Mann–Whitney U-test and Fisher’s exact test, for metric and nominal values, respectively

**Table 2:** Intraoperative and postoperative surgical parameters of patients undergoing DIFNG mastectomy, comparing TXA and no-TXA groups

	Total	TXA	No-TXA	*p* Value
No. of patients	75	41	34	–
Resection weight, g	980 [60, 3320]	909 [99, 3320]	1004 [60, 2948]	0.521
Drain output first three days, ml	140 [50, 360]	140 [65, 230]	165 [50, 360]	0.100
Total drain output, ml	150 [50, 400]	150 [65, 260]	167.5 [50, 400]	**0.048**
Drain duration, days	3 [2, 6]	3 [2, 5]	4 [3, 6]	**<0.001**
Hospital stay, days	3 [2, 6]	3 [2, 5]	4 [3, 6]	**0.008**
No. of follow-up visits	3 [1, 5]	3 [2, 5]	3 [1, 5]	0.151

Bold values statistically significant difference, *p* < 0.05

Values are represented by median [min, max]

Between-group differences were determined via Mann–Whitney U-tests

### Complications

In the overall study cohort, a total of 20 complications were recorded, with wound healing disorders being the most frequent. The number of complications per patient ranged from 0 to 2. Overall, no thromboembolic events, such as deep vein thrombosis, pulmonary embolism, and strokes, nor any seizures or hypotension were detected. Other commonly reported TXA-related adverse events, such as allergic skin reactions, gastrointestinal disturbances, or visual disturbances [21], were also not observed in this study.

In the TXA group, 8 complications were reported. Wound healing disorders, amounting to 12.2%, presented the most frequently reported adverse event, followed by seroma formation occurring in 4.9% of patients. Notably, no cases of postoperative bleeding requiring surgical revision were observed in this group. In the no-TXA group, the overall complication rate was higher, with 12 incidences in total. Seroma formation was the most common observed complication, affecting 14.7% of patients. Major bleeding events with surgical evacuation occurred significantly more frequent in the no-TXA group (11.8% vs 0%, *p* = 0.038). Further complications are summarized in Table 3.

**Table 3:** Postoperative complication rates following double incision mastectomy with free nipple grafts in patients with and without intraoperative TXA

	Total	TXA	No-TXA	*p* Value
No. of patients	75	41	34	–
No. of complications per patient	0 [0, 2]	0 [0, 1]	0 [0, 2]	0.377
Seroma formation	9.3% (7)	4.9% (2)	14.7% (5)	0.145
Bleeding	5.3% (4)	0.0% (0)	11.8% (4)	**0.038**
Wound healing disorder	10.7 % (8)	12.2 % (5)	8.8% (3)	0.466
Wound infection	1.3% (1)	2.4 % (1)	0.0% (0)	0.547
Surgical revision for hematoma evacuation	5.3% (4)	0.0% (0)	11.8% (4)	**0.038**

Bold values statistically significant difference, *p* < 0.05

Values are represented by median [min, max], percent (number)

Between-group differences were determined via Mann–Whitney U-test and Fisher’s exact test, for metric and nominal values, respectively

## Discussion

For many transgender males, gender-affirming mastectomy plays a central role in their transition to the affirmed gender [1, 2, 5], and ensuring patient safety while reducing the probability of revisional surgeries, often due to diffuse bleeding, is a key priority. The antifibrinolytic pharmacological agent TXA has therefore gained increased attention across multiple surgical specialties for its capacity to reduce perioperative blood loss and the need for postoperative transfusions [14, 27]. Numerous previously published studies have investigated the application of TXA in cosmetic and reconstructive procedures [14, 16, 28, 29]. To date, the literature on the administration of TXA in gender-affirming mastectomy remains limited, and documentation of drain duration and output is even more scarce [26]. Therefore, this study aimed to evaluate the impact of intravenous TXA in top surgery.

In comparison with other breast surgeries, gender-affirming mastectomy poses unique hemostatic challenges, owing to the significant removal of highly vascularized breast tissue and the formation of an extensive dead space [7]. Consequently, patients may be at increased risk of postoperative hematoma formation [7], which represents the most frequently reported complication in top surgery [12, 30–32]. This elevated risk may partially be explained by the relatively large unfilled dead space created after tissue resection, especially when compared to procedures such as autoaugmentation or breast reduction, where the space is occupied by a pedicle. In our study, the administration of TXA was associated with a complete absence of major bleeding-related complications, representing a significant reduction compared with the 11.8% rate observed in the no-TXA control group. This observation aligns with findings from Knight et al. in oncoplastic mastectomy [29], and Weissler et al., in implant-based breast reconstruction [15], both reporting significant reductions in hematoma rates with TXA utilization. These parallels are particularly relevant, as gender-affirming mastectomy shares fundamental procedural principles with these surgeries.

**Fig. 7:**
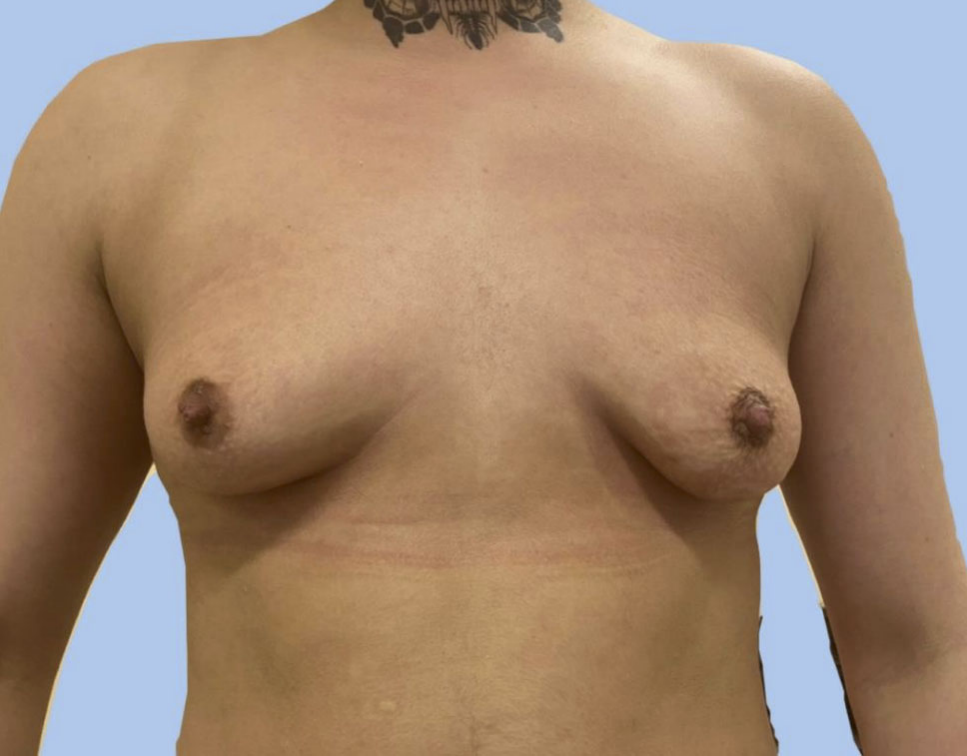
Preoperative frontal view of a 23-year-old transmasculine patient prior to top surgery, scheduled for DIFNG mastectomy

**Fig. 8:**
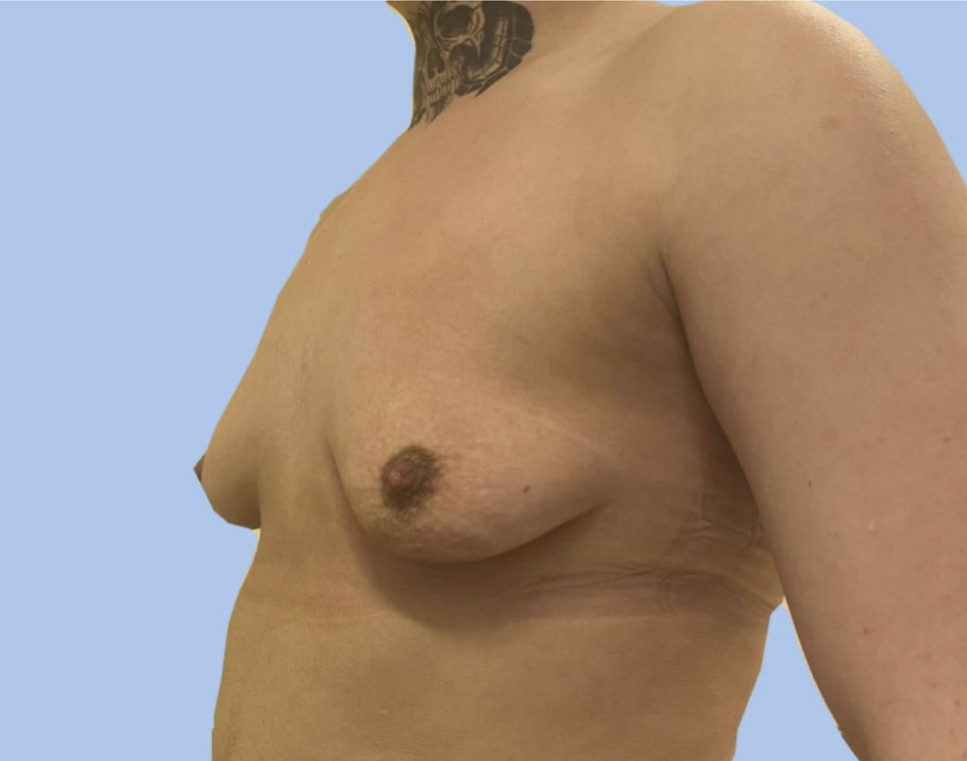
Preoperative 45-degree view of the same patient

**Fig. 9:**
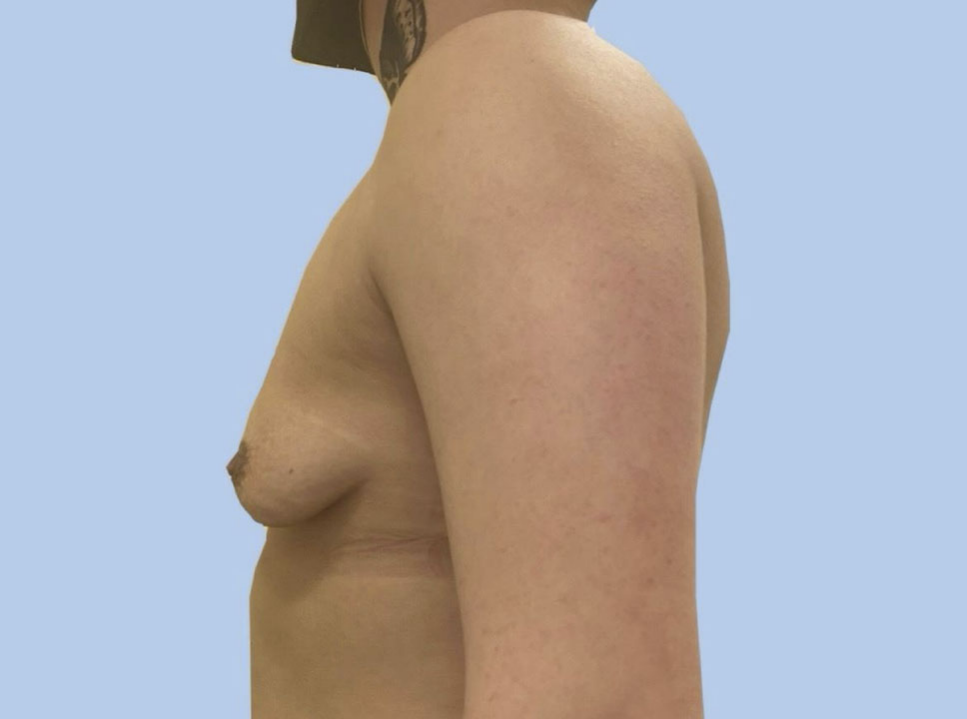
Preoperative lateral view of the same patient

**Fig. 10:**
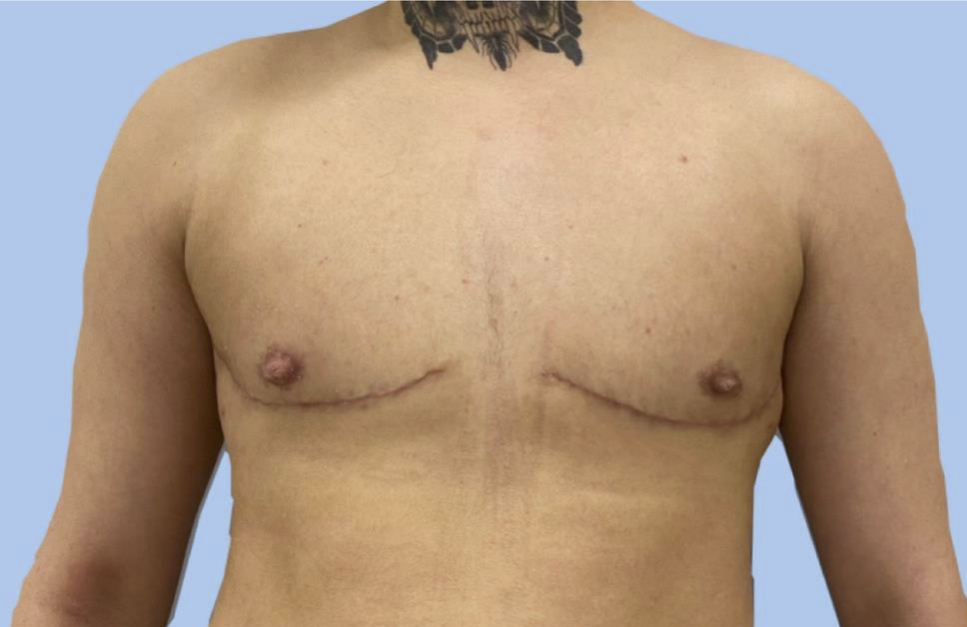
Postoperative frontal view of the same patient 3 months after a successfully performed DIFNG mastectomy

**Fig. 11:**
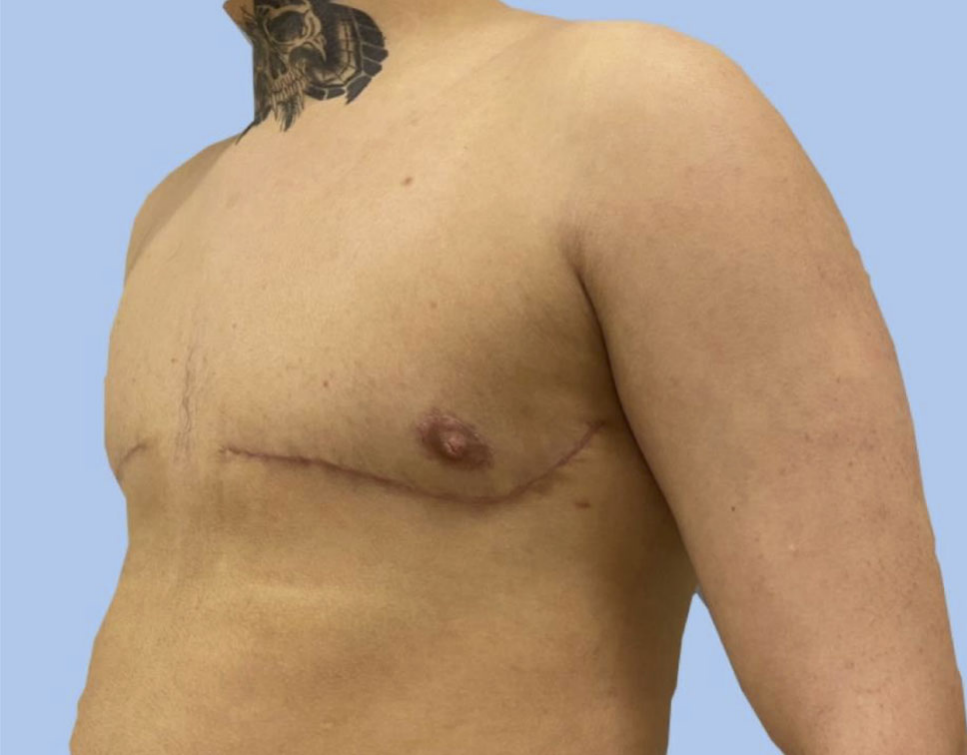
Postoperative 45-degree view of the same patient 3 months after surgery

**Fig. 12:**
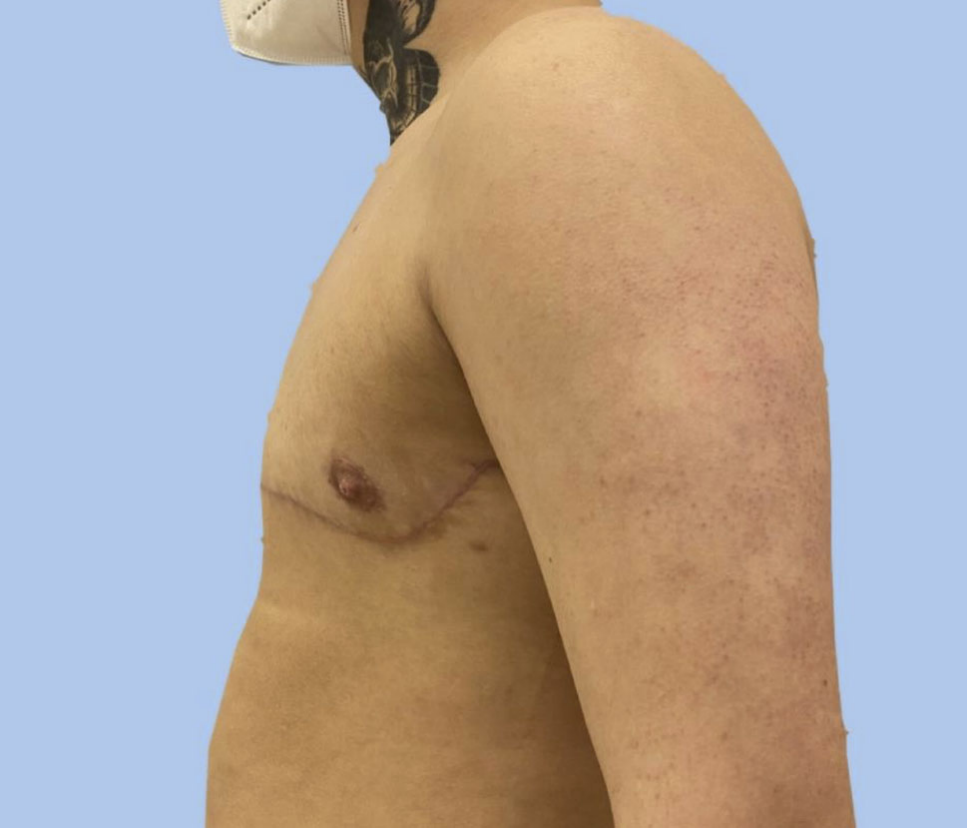
Postoperative lateral view of the same patient 3 months after surgery

Whether the surgical technique in top surgery affects hematoma formation remains inconclusive [7, 33]. Rifkin et al. reported higher hematoma rates after double incision procedures, whereas others identified increased postoperative bleeding potential in periareolar techniques due to limited visualization [12, 30, 33]. We believe these discrepancies indicate that factors such as distinct intraoperative hemostasis, intraoperative blood pressure management, and individual vascular anatomy may outweigh the surgical approach. Accordingly, we routinely elevate systolic blood pressure intraoperatively above 130 mmHg for several minutes to unmask minor bleeding sources before wound closure. Figures 7, 8 and 9 show an additional representative case of DIFNG mastectomy performed at our institution. Figures 10, 11 and 12 illustrate satisfactory postoperative outcomes.

Despite our promising results, the impact of intravenously administered TXA in other types of breast surgery, such as breast reduction, remains inconclusive to this day [34]. While Om et al. described a beneficial effect of TXA in breast reduction surgery, Weissler et al. found no significant advantage [17, 18]. We believe that such variations may partly stem from differences in outcome definition. Studies including both minor and major hematomas tended to report stronger effects than those focusing only on surgically relevant hematomas. In this study, minor hematomas were not systematically assessed, which may underestimate the overall effect of TXA. Future prospective studies are needed to provide a more complete characterization of TXA’s hemostatic profile.

TXA has been suggested to reduce exudative processes associated with the inflammatory response system [20] and thereby may influence seroma formation. Regarding the literature, seroma formation remains a frequently reported complication after gender-affirming mastectomy [31, 35]. This is consistent with our findings, and although seroma formation occurred in both cohorts, the incidence was markedly lower in the TXA group, suggesting a potential protective effect. While our group difference did not reach statistical significance, the observed trend supports this hypothesis. Various strategies have been proposed to minimize seroma risk, including meticulous intraoperative hemostasis, closed-suction drains, quilting or progressive tension sutures, and immobilization of the surgical site for a few days postoperatively [36, 37]. In our standardized surgical protocol, one closed-suction drain is placed per breast, followed by the use of immediate postoperative compression binders and a compression bra for 6 weeks. As a routine component of our postoperative management, drains are typically removed once the output is < 30 ml/24 h. This was consistent across both groups, making TXA a plausible contributing factor to the observed trend. The literature indicates that continuation of gender-affirming hormone therapy before top surgery is not associated with an increased risk of perioperative adverse events [38]. Similarly, all transmasculine patients in our study continued hormone therapy preoperatively.

A recent systematic review highlighted the lack of reported data on drain duration and output in studies examining TXA use in gender-affirming mastectomy, underscoring the need for further research [26]. To counter this literature gap, we defined these parameters as key outcome parameters. In this analysis, the TXA group demonstrated a significantly lower total drain output (*p* = 0.048), which correlated with a significant one day reduction in drainage duration (*p* < 0.001) and hospital stay (*p* = 0.008). Although the absolute reduction in total drain output between groups was relatively small, it reached statistical significance. At our institution, drain removal is guided by a predefined daily output threshold (< 30 ml/24 h) rather than by cumulative drainage volume. Small differences in postoperative drainage output may therefore translate into earlier drain removal, which is consistent with the significantly shorter drain duration, and reduced length of hospital stay observed in the TXA group. Furthermore, postoperative length of hospital stay should be interpreted in the context of local healthcare systems. Internationally, many plastic and reconstructive procedures are increasingly performed on an outpatient or short-stay basis [39], and in the USA in particular, same-day discharge after mastectomy procedures has become common [40]. Both cohorts in the present study were managed within the same institutional framework, allowing for a comparison of TXA-related outcomes. The observed significant reductions in drain duration, total drain output, and length of hospital stay may be directly linked to the reduced postoperative occurrence of seroma and hematoma formation in patients receiving TXA. Similar findings were described by Magni et al. in reduction mammaplasty, where TXA led to significantly lower drainage volumes and shortened length of hospital stay [41]. We believe that such improvements not only enhance patient comfort and recovery but may also improve overall surgical efficiency and postoperative outcomes. Although no consensus yet exists regarding the optimal dosage or administration of TXA in plastic surgery [14], our results support the efficacy and safety of weight adjusted intravenous TXA administration in DIFNG mastectomy.

Building on these results, we have routinely implemented the administration of intravenous TXA in patients undergoing DIFNG mastectomy. We believe that in addition to pharmacologic optimization, thorough preoperative counseling is essential to inform patients about individual bleeding tendencies, hematoma, and seroma risks. Notably, no TXA-related adverse events were observed, further supporting the safety of TXA administration in this setting.

### Limitations

This study has several limitations. The retrospective single-center design and the relatively small sample size may limit the generalizability of the findings. TXA administration was not randomized but based on an intraoperative surgeon’s judgment, introducing potential confounding by indication. Patients perceived to have a higher bleeding risk may have been more likely to receive TXA, reflecting real-world practice but limiting causal inference. Due to the retrospective design, limited sample size, and low number of major bleeding events, adjusted multivariable analyses were not performed. Moreover, variations in individual surgical techniques, patient anatomy, or perioperative management could have influenced complication rates despite standardized protocols. Minor hematomas not requiring clinical intervention were not systematically assessed. Therefore, the clinical effects of TXA may not be fully reflected by the evaluated endpoints. Future prospective, multicenter studies with standardized TXA dosing and outcome measures are warranted to validate these results.

## Conclusions

TXA is a well-established pharmacological agent for reducing perioperative blood loss. Despite its proven efficacy and safety, the current literature regarding its effectiveness in gender-affirming mastectomy remains limited. The outcome of this retrospective study is promising, as it indicates that the use of intravenously administered TXA contributes to a lower occurrence of postoperative seroma and hematoma formation in this surgical context. In addition, TXA was associated with a significantly lower drain output, drain duration, and length of hospital stay.

## Abbreviations

BMI: Body mass index
DIFNG: Double incision mastectomy with free nipple grafts
IMF: Inframammary fold
NAC: Nipple–areola complex
TXA: Tranexamic acid

## References

[1] Salibian AA, Gonzalez E, Frey JD, Bluebond-Langner R. Tips and tricks in gender-affirming mastectomy. Plast Reconstr Surg. 2021;147:1288–96. 10.1097/PRS.0000000000007997. 34019500 10.1097/PRS.0000000000007997

[2] Wright JD, Chen L, Suzuki Y, Matsuo K, Hershman DL. National estimates of gender-affirming surgery in the US. JAMA Netw Open. 2023;6:e2330348. 10.1001/jamanetworkopen.2023.30348. 37610753 10.1001/jamanetworkopen.2023.30348 PMC10448302

[3] Monstrey S, Selvaggi G, Ceulemans P, Van Landuyt K, Bowman C, Blondeel P, et al. Chest-wall contouring surgery in female-to-male transsexuals: a new algorithm. Plast Reconstr Surg. 2008;121:849–59. 10.1097/01.prs.0000299921.15447.b2. 18317134 10.1097/01.prs.0000299921.15447.b2

[4] Poudrier G, Nolan IT, Cook TE, Saia W, Motosko CC, Stranix JT, et al. Assessing quality of life and patient-reported satisfaction with masculinizing top surgery: a mixed-methods descriptive survey study. Plast Reconstr Surg. 2019;143:272–9. 10.1097/PRS.0000000000005113. 30286047 10.1097/PRS.0000000000005113

[5] Lane M, Kirsch MJ, Sluiter EC, Svientek SR, Hamill JB, Morrison SD, et al. Gender affirming mastectomy improves quality of life in transmasculine patients. Ann Surg. 2023;277:e725–9. 10.1097/SLA.0000000000005158. 34387203 10.1097/SLA.0000000000005158

[6] McEvenue G, Xu FZ, Cai R, McLean H. Female-to-male gender affirming top surgery: a single surgeon’s 15-year retrospective review and treatment algorithm. Aesthet Surg J. 2018;38:49–57. 10.1093/asj/sjx116. 10.1093/asj/sjx116 29040349

[7] Bluebond-Langner R, Berli JU, Sabino J, Chopra K, Singh D, Fischer B. Top surgery in transgender men: how far can you push the envelope? Plast Reconstr Surg. 2017;139:873e-e882. 10.1097/PRS.0000000000003225. 28350658 10.1097/PRS.0000000000003225

[8] Morselli PG, Summo V, Pinto V, Fabbri E, Meriggiola MC. Chest wall masculinization in female-to-male transsexuals: our treatment algorithm and life satisfaction questionnaire. Ann Plast Surg. 2019;83:629–35. 10.1097/SAP.0000000000002119. 31688107 10.1097/SAP.0000000000002119

[9] Cregten-Escobar P, Bouman MB, Buncamper ME, Mullender MG. Subcutaneous mastectomy in female-to-male transsexuals: a retrospective cohort-analysis of 202 patients. J Sex Med. 2012;9:3148–53. 10.1111/j.1743-6109.2012.02939.x. 23035854 10.1111/j.1743-6109.2012.02939.x

[10] Aristizábal A, Ríos-Sánchez M, Escandón JM, DeRoberts D, Armenta E, Del Corral G, et al. Body contouring as gender-affirming surgery in transgender patients: a systematic review of the current literature. J Clin Med. 2024;13:3523. 10.3390/jcm13123523. 38930052 10.3390/jcm13123523 PMC11204619

[11] Tolstrup A, Zetner D, Rosenberg J. Outcome measures in gender-confirming chest surgery: a systematic scoping review. Aesthetic Plast Surg. 2020;44:219–28. 10.1007/s00266-019-01523-1. 31664491 10.1007/s00266-019-01523-1

[12] Bekisz JM, Boyd CJ, Daar DA, Cripps CN, Bluebond-Langner R. Hematoma following gender-affirming mastectomy: a systematic review of the evidence. J Plast Reconstr Aesthet Surg. 2022;75:3108–21. 10.1016/j.bjps.2022.04.081. 35725957 10.1016/j.bjps.2022.04.081

[13] Frederick MJ, Berhanu AE, Bartlett R. Chest surgery in female to male transgender individuals. Ann Plast Surg. 2017;78:249–53. 10.1097/SAP.0000000000000882. 27845966 10.1097/SAP.0000000000000882

[14] Scarafoni EE. A systematic review of tranexamic acid in plastic surgery: what’s new? Plastic Reconstruct Surg Global Open. 2021;9:e3172. 10.1097/GOX.0000000000003172. 10.1097/GOX.0000000000003172 PMC8062149 33907653

[15] Weissler JM, Banuelos J, Jacobson SR, Manrique OJ, Nguyen M-DT, Harless CA, et al. Intravenous tranexamic acid in implant-based breast reconstruction safely reduces hematoma without thromboembolic events. Plast Reconstr Surg. 2020;146:238–45. 10.1097/PRS.0000000000006967. 32740567 10.1097/PRS.0000000000006967

[16] Rohrich RJ, Brown S, Brown T, Taub PJ. Role of tranexamic acid (TXA) in plastic and reconstructive surgery: a national perspective. J Plast Reconstr Aesthet Surg. 2024;102:373–83. 10.1016/j.bjps.2024.09.085. 39965470 10.1016/j.bjps.2024.09.085

[17] Weissler JM, Kuruoglu D, Antezana L, Curiel D, Kerivan L, Alsayed A, et al. Efficacy of tranexamic acid in reducing seroma and hematoma formation following reduction mammaplasty. Aesthet Surg J. 2022;42:616–25. 10.1093/asj/sjab399. 35029651 10.1093/asj/sjab399

[18] Om A, Marxen T, Kebede S, Losken A. The usage of intravenous tranexamic acid in reduction mammaplasty safely reduces hematoma rates. Ann Plast Surg. 2023;90:S371–4. 10.1097/SAP.0000000000003296. 36729851 10.1097/SAP.0000000000003296 PMC10578999

[19] Zaussinger M, Kerschbaumer C, Schwartz B, Bachleitner K, Ehebruster G, Schmidt M. Influence of tranexamic acid in body contouring surgery: significant changes on complication rates after abdominoplasty. Aesthetic Plast Surg. 2024;48:2872–8. 10.1007/s00266-024-04094-y. 38750226 10.1007/s00266-024-04094-y

[20] Jimenez JJ, Iribarren JL, Lorente L, Rodriguez JM, Hernandez D, Nassar I, et al. Tranexamic acid attenuates inflammatory response in cardiopulmonary bypass surgery through blockade of fibrinolysis: a case control study followed by a randomized double-blind controlled trial. Crit Care. 2007;11:R117. 10.1186/cc6173. 17988379 10.1186/cc6173 PMC2246206

[21] Ng W, Jerath A, Wasowicz M. Tranexamic acid: a clinical review. Anaesthesiol Intensive Ther. 2015;47:339–50. 10.5603/AIT.a2015.0011. 25797505 10.5603/AIT.a2015.0011

[22] Falade I, Lopes A, Switalla K, Song S, Ramrakhiani N, Kim E. Efficacy of topical tranexamic acid in gender-affirming mastectomy. J Plast Reconstr Aesthet Surg. 2025;102:255–61. 10.1016/j.bjps.2025.01.048. 39947111 10.1016/j.bjps.2025.01.048

[23] Sipos K, Joensuu K, Kauhanen S, Ojala K. Topical tranexamic acid and chest masculinization surgeries—impact on postoperative hematoma incidence. JPRAS Open. 2025;43:458–69. 10.1016/j.jpra.2025.01.002. 39989714 10.1016/j.jpra.2025.01.002 PMC11847029

[24] Rifkin WJ, Parker A, Bluebond-Langner R. Use of tranexamic acid in gender-affirming mastectomy reduces rates of postoperative hematoma and seroma. Plast Reconstr Surg. 2024;153:1002e-e1010. 10.1097/PRS.0000000000010892. 37399532 10.1097/PRS.0000000000010892

[25] Edalatpour A, Seitz AJ, Warden AM, Gunderson K, Wirth PJ, Rose K, et al. Outcomes of enhanced recovery protocols and tranexamic acid on double-incision versus periareolar gender-affirming mastectomy: A retrospective study of postoperative outcomes. J Plast Reconstr Aesthet Surg. 2024;88:360–8. 10.1016/j.bjps.2023.11.027. 38061259 10.1016/j.bjps.2023.11.027

[26] Fung E, Shaari D, Li A, Yu BZ, Roth JM, Taub PJ. The use of tranexamic acid in gender-affirming mastectomy: a systematic review and meta-analysis. Eur J Plast Surg. 2025;48:77. 10.1007/s00238-025-02336-z.

[27] Brown S, Yao A, Taub PJ. Antifibrinolytic agents in plastic surgery: current practices and future directions. Plast Reconstr Surg. 2018;141:937e-e949. 10.1097/PRS.0000000000004421. 29794717 10.1097/PRS.0000000000004421

[28] Laikhter E, Comer CD, Shiah E, Manstein SM, Bain PA, Lin SJ. A systematic review and meta-analysis evaluating the impact of tranexamic acid administration in aesthetic plastic surgery. Aesthet Surg J. 2022;42:548–58. 10.1093/asj/sjab333. 34486647 10.1093/asj/sjab333

[29] Knight H, Banks J, Muchmore J, Ives C, Green M. Examining the use of intraoperative tranexamic acid in oncoplastic breast surgery. Breast J. 2019;25:1047–9. 10.1111/tbj.13409. 31187540 10.1111/tbj.13409

[30] Cohen WA, Shah NR, Iwanicki M, Therattil PJ, Keith JD. Female-to-Male transgender chest contouring. Ann Plast Surg. 2019;83:589–93. 10.1097/SAP.0000000000001896. 31082837 10.1097/SAP.0000000000001896

[31] Rothenberg KA, Gologorsky RC, Hojilla JC, Tang A, Cohan MC, Beattie G, et al. Gender-affirming mastectomy in transmasculine patients. Ann Plast Surg. 2021;87:24–30. 10.1097/SAP.0000000000002712. 33559996 10.1097/SAP.0000000000002712 PMC8936918

[32] Ederer IA, Spennato S, Nguyen C-T, Wehle A, Wachtel C, Kiehlmann M, et al. A single-center 10-year experience of 180 transmasculine patients undergoing gender-affirming mastectomy while continuing masculinizing hormone replacement therapy. Aesthetic Plast Surg. 2023;47:946–54. 10.1007/s00266-022-03213-x. 36510021 10.1007/s00266-022-03213-x

[33] Rifkin WJ, Robinson IS, Kloer C, Cripps CN, Boyd CJ, Blasdel G, et al. Gender-affirming mastectomy: comparison of periareolar and double incision patterns. Plast Reconstr Surg Glob Open. 2022;10:e4356. 10.1097/GOX.0000000000004356. 35646495 10.1097/GOX.0000000000004356 PMC9132529

[34] Pontes A, Barreiro D, Costa-Ferreira A. The impact of tranexamic acid administration in reduction mammaplasty. Ann Plast Surg. 2025;94:370–7. 10.1097/SAP.0000000000004184. 39787391 10.1097/SAP.0000000000004184

[35] Cordero JJ, Alaniz L, Kalavacherla S, Kadakia N, Machol JA, Carré AL, et al. Review of gender affirming mastectomy surgery. Ann Plast Surg. 2024;93:308–11. 10.1097/SAP.0000000000004037. 39158332 10.1097/SAP.0000000000004037

[36] Janis JE, Khansa L, Khansa I. Strategies for postoperative seroma prevention: A systematic review. Plast Reconstr Surg. 2016;138:240–52. 10.1097/PRS.0000000000002245. 27348657 10.1097/PRS.0000000000002245

[37] Morarasu S, Clancy C, Ghetu N, Musina AM, Velenciuc N, Iacob S, et al. Impact of quilting sutures on surgical outcomes after mastectomy: A systematic review and meta-analysis. Ann Surg Oncol. 2022;29:3785–97. 10.1245/s10434-022-11350-5. 35103890 10.1245/s10434-022-11350-5

[38] Wu SS, Raymer CA, Kaufman BR, Isakov R, Ferrando CA. The effect of preoperative gender-affirming hormone therapy use on perioperative adverse events in transmasculine individuals undergoing masculinizing chest surgery for gender affirmation. Aesthet Surg J. 2022;42:1009–16. 10.1093/asj/sjac091. 35417528 10.1093/asj/sjac091

[39] Zhang KK, Reddy N, Janis JE. Office-based plastic surgery—evidence-based clinical and administrative guidelines. Plast Reconstr Surg Glob Open. 2022;10:e4634. 10.1097/GOX.0000000000004634. 36381487 10.1097/GOX.0000000000004634 PMC9645793

[40] Sibia US, Klune JR, Turcotte JJ, Holton LH, Riker AI. Hospital-based same-day compared to overnight-stay mastectomy: an American College of Surgeons National Surgical Quality Improvement Program analysis. Ochsner J. 2022;22:139–45. 10.31486/toj.21.0103. 35756587 10.31486/toj.21.0103 PMC9196968

[41] Magni S, Guggenheim L, Fournier G, Parodi C, Pagnamenta A, Schmauss D, et al. The effects of systemic tranexamic acid administration on drainage volume, length of hospital stay, and postoperative complications in reduction mammaplasty. J Clin Med. 2024;14:151. 10.3390/jcm14010151. 39797232 10.3390/jcm14010151 PMC11720834

